# Response of Elementary Structural Transitions in Glassy Atactic Polystyrene to Temperature and Deformation

**DOI:** 10.1021/acs.jpcb.2c04199

**Published:** 2022-09-21

**Authors:** Georgios G. Vogiatzis, Lambèrt C.A. van Breemen, Markus Hütter

**Affiliations:** † Polymer Technology, Department of Mechanical Engineering, Eindhoven University of Technology, P.O. Box 513, 5600 MB Eindhoven, The Netherlands; ‡ Dutch Polymer Institute, P.O. Box 902, 5600 AX Eindhoven, The Netherlands

## Abstract

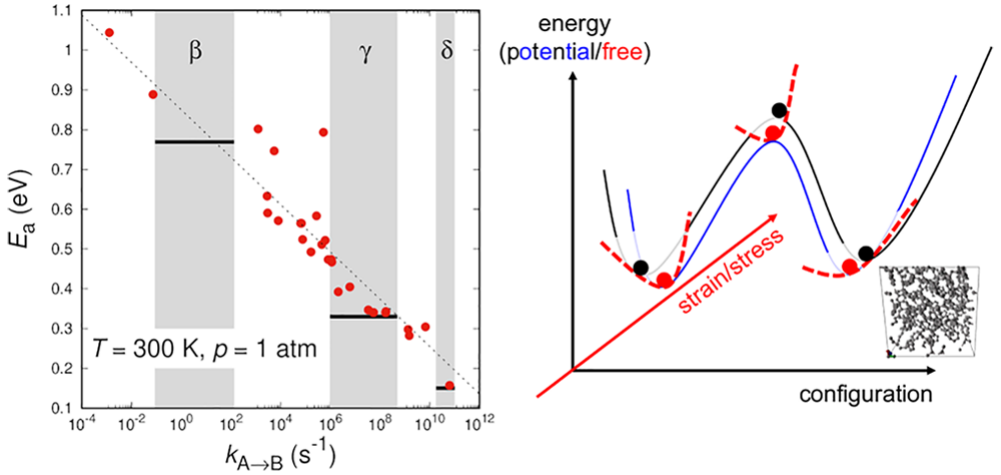

The effects of temperature, pressure, and imposed strain on the structural transition pathways of glassy atactic polystyrene (aPS) are studied for a wide range of conditions. By employing an atomistic description of the system, we systematically explore its free energy landscape, emphasizing connections between local free energy minima. A triplet of two minima connected to each other via a first-order saddle point provides the full description of each elementary structural relaxation event. The basis of the analysis is the potential energy landscape (PEL), where efficient methods for finding saddle points and exploring transition pathways have been developed. We then translate the stationary points of the PEL to stationary points of the proper free energy landscape that obeys the macroscopically imposed constraints (either stress- or strain-controlled). By changing the temperature under isobaric conditions (i.e., Gibbs energy landscape), we probe the temperature dependence of the transition rates of the subglass relaxations of aPS, thus obtaining their activation energies by fitting to the Arrhenius equation. The imposition of different strain levels under isothermic conditions allows us to estimate the apparent activation volume of every elementary transition. Our findings are in good agreement with experimental observations for the same system, indicating that both length- and time-scales of the structural transitions of glassy aPS can be obtained by proper free energy minimization of atomistically detailed configurations.

## 1 Introduction

The study of the molecular origin of the time-dependent properties of (polymer) glasses is still a mostly unsolved and open problem. Polymer glasses are extensively used in a wide range of applications and produced in enormous quantities from commodity-rated packaging thin films all the way to extremely pure medical-grade substances and blends. Apart from the industrial interest, the dynamics of the glassy state is still elusive due to the long time-scales involved. One of the most prominent approaches to the problem is by employing a mapping of the glassy configurations to the underlying potential energy landscape (PEL) of the system and study the motion of the system between the subspaces of the landscape.^1,2^ A natural way to tessellate the energy landscape is by means of “basins of attraction” around local potential energy minima (also called “inherent structures” following Stillinger and Weber^3,4^). Each basin engulfs a specific minimum (or even multiple minima, e.g., double wells), and any steepest descent path initiated within the basin will lead to the local minimum at its bottom. Within the PEL picture, dynamics is governed by jumps from a basin to its neighboring basins. A reasonable approximation to studying the time-dependent properties of glasses is to split the problem in estimating the sought property at the minima (where the system spends most of the time) and combine it with the time evolution which is governed by the transition states (configurations at the top of the ridges separating basins).

Theodorou and Suter were the first ones to study the mechanical properties of polymer glasses by means of the response to deformation of specific local minima of the PEL.^5^ They envisioned that energetically stable configurations (i.e., local minima of the potential energy landscape) contribute primarily to the elastic behavior of the glasses observed macroscopically. By assuming, on the basis of a thermodynamic analysis, that the entropic contribution to the elastic constants of their model polypropylene (PP) glass was minute compared to the energetic one, they managed to calculate the elastic constants by monitoring the change in the potential energy (i.e., equivalent to the free energy as *T* → 0) of the specimens when subjected to infinitesimal deformations. Complementary to the energetic approach, they employed the virial theorem approach with microscopically calculated stresses (which is fully equivalent to solving the force and torque balance equations for all atoms within the system) in order to extract the elastic constants of glassy PP. The agreement with experimental measurements was remarkable (within 15% at most).

While the study of the energy minima can provide estimates of the structural and thermodynamic properties of the system, dynamics is governed by the existence of transition states. By the term transition states we define first-order saddle points connecting two different basins, centered around local minima on the potential energy landscape. While they system may travel through higher-order saddle points, we limit ourselves to those that have a single negative eigenvalue. This is definitely an assumption with the purpose of rigorously defining a single path between two basins and alleviating part of the computational burden to discover higher-order saddle points. Starting out of a minimum (that can be easily reached starting from any point in its basin), it is challenging to find transition states in the surroundings.

In our previous work,^6^ we formulated a method (by combining and extending earlier approaches to the problem) for carefully stepping on the rugged energy landscape of glassy polymers described at an atomistic resolution with classical molecular force fields. We split the whole process in three steps, i.e., exiting the convex region of the basin surrounding the initial minimum, approaching the transition state, and exploring the minimal energy path (intrinsic reaction coordinate (IRC) path following Fukui^7,8^) that connects two neighboring minima. In the course of the process, a transition state and a new local minimum are discovered.

The method for locating saddle points proceeds by (a) stepping out of the minimum by following a specific direction in phase-space (this direction can be parallel to an eigenvector of the Hessian, a linear combination of specific modes of the Hessian, a random direction in space or a local excitation in the sense of Mousseau and Barkema^9^), (b) projecting the potential energy gradient of the system on the step vector (by means of the transformation proposed by Munro and Wales^10^), and (c) minimizing the potential energy in the normal directions with respect to the proposed step. Once the system finds itself out of the convex region, we used the method of Baker^11^ to drive it to a first-order saddle point. As far as the construction of the IRC path is concerned, we employed the method of Page and McIver^12^ that is based on a local quadratic approximation (LQA) to the potential energy landscape. The whole process of discovering saddle points and exploring the IRC is greatly facilitated by exploiting rigorous and computationally tractable analytical derivatives for the potential energy.^13^

The free energy of the polymeric specimen at the stationary points (local minima and transition states) of the PEL is calculated by applying the quasi-harmonic approximation to the Helmholtz energy, as elaborated in ref (13). In essence, the free energy under constant shape of the simulation box (i.e., its Helmholtz energy) can be obtained as the sum of the potential energy at the stationary point and a vibrational contribution calculated by treating all degrees of freedom as independent (classical or) quantum harmonic oscillators, whose frequencies are obtained from the eigenvalues of the Hessian in mass-weighted coordinates. Starting from the Helmholtz energy and by cautiously applying the Legendre transform, we can produce the free energy functional for any combination of stresses and strains imposed on the simulation box. Minimization of a properly defined free energy functional for uniaxial compression or extension allowed us to mimic the macroscopic tensile testing experiments. By adopting a description with two independent parameters (ε, σ_⊥_) and the assumption of a shape-preserving deformation in the lateral directions (i.e., ε_*yy*_ = ε_*zz*_ = ε_⊥_ for ε = ε_*xx*_, and similarly for ε = ε_*yy*_ and ε = ε_*zz*_), the problem of simulating a uniaxial deformation experiment became computationally tractable. The deformation involved the application of extremely small steps in strain, while minimizing the relevant free energy function of every configuration with respect to the externally imposed stress constraints (quasi-static deformation) in the lateral directions. While it could not incorporate the effect of a finite strain-rate, the deformation protocol provided us with a wealth of information for the response of the system in the elastic^14^ and the plastic regimes.^13^

The imposition of deformation destabilizes the local potential energy minima. Even within the elastic regime, plastic events^15^ can be discerned where the system drifts from one minimum to another.^16^ The basin of the original minimum is distorted to such an extent that it is no longer convex. Within the framework set by Eyring,^17^ the energy barriers separating minima decrease linearly with strain facilitating thermally activated transitions. Lacks and co-workers^18−20^ showed that plastic deformation induced by shearing causes the disappearance of local potential energy minima which have been destabilized along a single zero-mode. More recently, Chung and Lacks^21^ stated that the disappearance of minima due to plastic deformation is equivalent to the mathematical “fold catastrophe”; i.e., one of the two minima connected to a saddle point is forced to merge with the saddle point, so that both the minimum and the saddle point disappear. Moreover, during this process the curvature of the minimum flattens out with a well-defined scaling behavior.^22^

In this work, we report results on the quasi-static deformation of transition states on the energy landscape of atactic polystyrene at room temperature. In order to study the effect of temperature and pressure, we explore the Gibbs energy landscape. The latter is accomplished by searching for free energy minima close to the stationary points of the potential energy landscape; i.e., we employ the quasi-harmonic approximation to estimate the relevant Gibbs energy at the local minima and first-order saddle points of the potential energy landscape (whose exploration has been presented in ref (6)). All derivatives can be obtained by rigorous analytic expressions presented in ref (13). The combination of the above methods allows us to microscopically mimic a macroscopic equilibrium under given pressure, strain, or stress. The study of connected triplets of local minima and transition states (first-order saddle points) enables us to study for the first time the dependence of the corresponding rate constants on temperature, pressure, and applied strain. Extending the study of the potential energy minima initiated by Theodorou and Suter^5^ to stationary points of a properly defined free energy reveals a wealth of important observations and paves the way to direct connections to experimental observables.

## 2 Methodology

### 2.1 Microscopic Model

We consider a microscopic specimen of a glassy material that is represented at the atomistic scale; i.e., it consists of discrete (united) atoms interacting via a classical molecular force field. Thus, the degrees of freedom of our description are the Cartesian coordinates of all atoms, i.e., the set {**r**_*i*_} and a suitable measure of the imposed deformation, either strain, ε_*κλ*_ (or deformation gradient), or stress (σ_*κλ*_). Combinations of strain- and stress-controlled boundary conditions are also possible.^13^ In what follows, we employ the convention of using bold symbols for representing full tensorial or vector quantities (e.g., **r**_*i*_), while individual elements of vectors or tensors are represented by appropriate regular Greek indices, e.g., ε_*κλ*_. Latin subscripts, e.g., *i* in **r**_*i*_, are reserved for indexing atoms. As shown in our earlier studies, the adoption of a classical molecular force field allows for analytical treatment of its first- and second-order derivatives with respect to the atomic positions and deformation measures (e.g., strain or deformation gradient).^13,23^

In the standard energy landscape picture of the glassy state, the system is mostly found within basins of the energy landscape surrounding local minima.^2^ From time to time, the system may move from one basin to another via infrequent jumps over transition states (first-order saddle points of the potential energy). The jumping process involves the system going uphill and downhill through a valley (minimal energy path) connecting the two minima through the saddle point. For studying the dependence of glassy dynamics on deformation, we should focus on calculating the rate constants for these individual jumps. This is accomplished by employing the multidimensional transition state theory (TST).^24^ However, application of TST hinges upon a proper calculation of the free energy of the specimen (under the externally imposed constraints) at the local minima and first-order saddle points of the energy landscape.

In the following, we study the free energy landscape of a glassy atactic PS specimen in a united-atom representation (hydrogen atoms are fused to the carbon atoms that are chemically attached). We have extensively used the molecular model of Lyulin and Michels^25^ in the past, and we employ it for the present study, too. The simulation box consists of four PS chains, whose molecular weight is 30 kg/mol and the styrene dyads (meso or racemo) follow a Bernoullian distribution with mean 0.5. Initial configurations have been prepared by using the two-scale equilibration protocol presented in ref (26). PS has been the material of choice due to its fast physical aging kinetics; its glass properties have been recovered successfully by molecular simulations.^6,13,25^ The methods are applicable to any atomistically detailed system described by a classical force field; there is no limitation to the chemical structure.

### 2.2 Helmholtz Energy at Stationary Points of the Potential Energy Landscape

Following the extensive literature on the quasi-harmonic approximation (QHA) to the free energy,^5,13,14,24,27^ the Helmholtz energy of the system located within a basin *I*, *A*_*I*_, can be readily obtained as
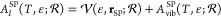
1where we have introduced the notation “SP” to indicate that the Helmholtz energy is calculated at a stationary point of the potential energy. It is to be noted that, in the Helmholtz energy given by eq 1, only the vibrational contribution depends on temperature. It is split into the potential energy, , at atomic positions **r** = **r**_SP_, while the Helmholtz energy of the *N*_DOFs_ = 3*N* – 3 vibrational modes^5,28,29^ is denoted by *A*_vib_ and is calculated for the configuration of the system at the stationary point. The strain appearing in eq 1 refers to the Cauchy strain tensor. In our simulations, we employ a rectangular parallelpiped simulation box. However, the calculation of the Helmholtz energy and its derivatives to be presented in the following section is applicable to system of any geometry (e.g., monoclinic or triclinic) given that the calculation of interatomic separation vectors within the minimum image convention is accomplished by correctly treating the periodic bondary conditions.^30^ The quasi-harmonic approximation limits the temperature range of applicability of our approach, but it remains a valid approximation even at elevated temperatures. The interested reader is referred to a previous study^14^ where the QHA was validated against experimental data and results from molecular dynamics simulations.

Since we have assumed that the system is located at a stationary point of the potential energy landscape, its potential energy around that point can be approximated by a Taylor expansion
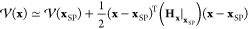
2in terms of the mass-weighted atomic coordinates, **x**_*a*_ = *m*_*a*_^1/2^**r**_*a*_, which is truncated after the second-order term.^5^ There is no first-order term (potential energy gradient) since we are treating stationary points and the second-order term is governed by the 3*N* × 3*N* Hessian matrix (i.e., second derivatives) of the potential energy with respect to all mass-weighted coordinates
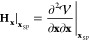
3If the systems exhibit any kind of symmetry (e.g., translational or rotational invariance), the Hessian should be transformed in order to exclude this symmetry, leading to a matrix with an effective range of *N*_DOFs_ × *N*_DOFs_ elements.

The Hessian matrix provides us with a way to calculate the vibrational contribution to the free energy. The eigenvalues, ω_*j*_^2^, and the eigenvectors, **v**_*j*_, of the Hessian correspond to the vibrational frequencies and the normal modes, respectively, of the motion of the system within the basin, if this is assumed to be the superposition of *N*_DOFS_ independent uncorrelated harmonic oscillators.^27^ By assuming that the oscillators have discrete energy levels, i.e., a quantum-mechanical treatment holds, the vibrational contribution to the Helmholtz energy is calculated as^24,31^
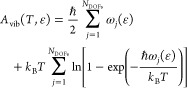
4with *k*_B_ as the Boltzmann constant and ℏ = *h*/(2π), with *h* being Planck’s constant. By following classical thermodynamics, the Helmholtz energy is split as *A* = *U* – *TS*, where *S* is the entropy of the microscopic system^32,33^
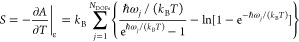
5and *U* is the internal energy^13^
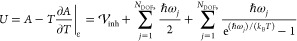
6Both entropy and internal energy can be calculated from the potential energy of the stationary point and the vibrational frequencies obtained there.

### 2.3 Deformation Thermodynamics

Following the free energy minimization technique with respect to the components of the strain tensor (with analytic derivatives of the potential energy both with respect to Cartesian coordinates, **r**_*i*_ and components of the strain tensor, ε_*κλ*_) we introduced in our previous work,^13^ any deformation to the system will be applied in a stepwise fashion of strain steps on the order of . Each step of the deformation can be adequately treated within the infinitesimal strain theory; i.e., we are employing the Cauchy strain, ε, as the measure of the deformation.

We consider a rectangular parallelpiped simulation box, with edge vectors **h**_1_, **h**_2_, **h**_3_, encompassing *N* interacting atoms. The tensor  is formed by combining the edge vectors of the periodic simulation box. We have shown^13^ that infinitesimal strains ε induce a change in the box dimensions, δ**h** = **h** – , that is a small perturbation relative to the box dimensions at the reference configuration , . The Cauchy strain is then given by
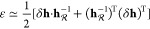
7In the process, we have discarded all terms equal or to higher than second-order in δ**h**, given that we are interested in infinitesimal deformations, and we end up with a symmetric linear strain tensor. Furthermore, by employing a rectangular parallelpiped simulation box (that is convenient for simulations of amorphous systems),  is diagonal and so is , and eq 7 can be simplified:
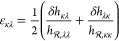
8

The differential of the Helmholtz energy, in the limit of infinitesimally small strains, is given by
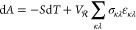
9where the Cauchy stress tensor is identified as
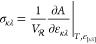
10where the notation [*κλ*] is employed to indicate that all elements of **ε** except ε_*κλ*_ are held constant during differentiation.^34^ The Helmholtz energy per unit volume, *A*/*V*, is usually termed as the elastic energy function in the field of linear elasticity.^35^

### 2.4 Free Energy of a Specimen under Uniaxial Deformation

In order to mimic the macroscopic uniaxial deformation experiments, we should use a suitable Legendre transform of the Helmholtz energy, where the size of the simulation box is allowed to change in one direction, while ambient pressure is applied to the lateral ones. A proper free energy functional should, on one hand, allow our method to be able to mimic macroscopic experiments, while on the other hand, be based on the fewest thermodynamic variables in order to make the problem computationally tractable. By imposing a strain-controlled deformation along one direction, fully described by the strain in that direction ε = ε_∥_, while assuming that the deformation in the lateral directions is isotropic and controlled by a lateral stress, σ_⊥_, we can derive a free energy functional which depends only on ε.^13^

By excluding the off-diagonal components of the strain tensor, eq 9 becomes
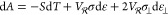
11where σ and σ_⊥_ are the stresses in the principal and the lateral directions of the deformation, respectively. By introducing the volumetric change ϕ = 1 + ε + 2ε_⊥_, we can rewrite eq 11 in terms of ε and ϕ:
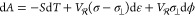
12where differentials of ε and ϕ appear. We can now define a Legendre transform of *A* with respect to ϕ, where the stress applied to the lateral directions σ_⊥_ will replace ϕ:
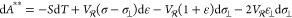
13This gives us the fundamental equation in the A** representation:
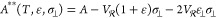
14A** is a function of the imposed strain in the one direction, ε, and the stress applied to the equally deformed cross-section in the lateral directions, σ_⊥_. We used the double-asterisk-notation, A**, to discern our free energy functional that preserves the shape of the cross-section, from a Legendre transform of the form  where some components of the strain tensor have been replaced by components of the stress tensor. Thermodynamic equilibrium of the A** potential implies that it is minimal with any change of the lateral size of the system, under imposed *T*, ε, and σ_⊥_. We note here that eq 14 is equivalent to the small-strain limit of the A** free energy derived in eq 37 of ref (13).

### 2.5 Free Energy of a Specimen under Imposed Stress

In the case of an elastic solid, there is no unique Gibbs energy; Li et al.^36^ provided a thorough analysis on the concept of the chemical potential of an elastically stressed solid. They showed that a free energy function whose partial derivative with respect to the number of moles would yield a proper chemical potential does not exist. There are few exceptions, e.g., the case of purely hydrostatic stress where McLellan derived a chemical potential assuming thermomechanical equilibrium,^37^ but generally, a solid specimen can be characterized only by a properly defined entropy *S*, and Helmholtz energy, *A*, which depend on strain and temperature as discussed above. In the following, we are going to consider quantities analogous to the Gibbs energy, *G*, and enthalpy, *H*.^38^

By starting from the fundamental equation for *A* in differential form, eq 9, we can formulate a new thermodynamic potential that has the temperature and the stress tensor as independent variables, *G*(*T*, **σ**), as the Legendre transform of *A* with respect to the components of a strain tensor whose diagonal elements have been increased by 1/3, i.e.
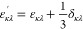
15with δ_*κλ*_ being the Kronecker delta. Since the constant 1/3 added to the diagonal components of the strain tensor does not contribute to the differential, we can have the following Legendre transform of *A* based on eq 9:
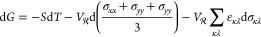
16By introducing the hydrostatic pressure, *p*=–(1/3)Tr(**σ**), we can rewrite eq 16 as
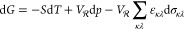
17This substitution brings us to a Gibbs energy definition for our solid specimen:
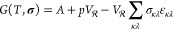
18that resembles the Gibbs energy of a fluid. It is in the spirit of previous derivations by Morris^39^ and Lempesis et al.^14^ If we had not included the factor 1/3 in the differential, we would end up with the definition of the complementary energy function of the theory of elasticity
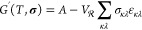
that is also frequently referred to as Gibbs energy. The definitions of *G* and *G*′ differ by the term  that is constant with respect to the independent parameters of the free energy; they are therefore equivalent since only differences in free energy matter. However, we prefer to coin the term “Gibbs” energy for the free energy of eq 18 that resembles the Gibbs energy function of a fluid. Based on the generalized energy functions A** and *G* defined above, a consistent thermodynamic framework for calculating deformation-dependent transition rates is developed.

### 2.6 Locating Stationary States on the Potential Energy Landscape

While it is rigorous to discover potential energy local minima by means of efficient minimization methods, the problem of discovering first-order saddle points that can serve as transition states is still unsolved. In our previous work,^6^ we suggested a combination of methods for sampling first-order saddle points, conceptually similar to the eigenvector-following technique of Munro and Wales^10^ and the activation-relaxation technique (ART) of Barkema and Mousseau.^9,40^ Our proposed approach that follows randomly chosen eigenvectors of the Hessian was successful in probing the elementary structural transitions on the potential energy landscape of a united-atom atactic PS specimen. We have also tested the local random excitation of a group of atoms that is at the basis of the ART method; however, it proved less efficient in our systems of interest. The main difference between the two classes of methods is the spatial extent and intensity of the excitation: the ART method excites a specific group of atoms (while leaving all other unperturbed at its first step), while the eigenvector-following method excites all atoms along a vibrational mode of the system. While there are no clear advantages to either approach, we preferred using the eigenvector-following one, enriched by linear combinations of eigenvectors of the Hessian as initial directions out of a minimum (increasing the level of randomness and complexity in the choice of the initial search direction). Since it serves as the basis for exploring the free energy landscape of our specimens, we briefly present its main components in the following paragraphs.

At a local minimum, where a diagonalization of the Hessian has taken place, we randomly choose one of its eigenvectors to follow and the system is stepwise translated from the minimum along the valley floor parallel to the chosen normalized eigenvector 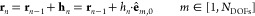
19with *h*_*n*_ being the magnitude of the step vector at step *n* and  the eigenvector corresponding to the *m*-th eigenvalue calculated at the minimum (i.e., step “0”). As discussed above, the dimensionality of the Hessian matrix is *N*_DOFs_ × *N*_DOFs_ after excluding any potential symmetries of the system (in our case translational invariance). We sample the eigenvector space (i.e., possible orientations for the saddle point searching path) randomly to avoid introducing any bias to the distribution of saddle points to be discovered (e.g., by following the lower modes of the Hessian). At every step, we maximize the potential energy in the “walking” direction parallel to the streambed we are following while seeking a minimal potential energy with respect to all other lateral directions.^10^ The magnitude of the step-size can be arbitrarily chosen, but we subject it to a backtracking process so that the uphill walk remains as close to the underlying streambed as possible. When the system escapes from the convex region of the basin surrounding a local minimum, we exchange the stepping method with the method of Baker for following the lowest eigenmode of the Hessian,^41^ until the system converges to a first-order saddle point. The interested reader is referred to ref (6) for a detailed presentation.

Out of the saddle point we construct a minimal energy path following the intrinsic reaction coordinate (IRC) construction of Fukui.^8^ The IRC is an imaginary trajectory in the mass-weighted Cartesian space that goes infinitely slowly through the transition state. The trajectory conncets two neighboring minima through a transition state. Close to the transition state, it is parallel to the eigenvector corresponding to the single negative eigenvalue of the Hessian. As in our previous work,^6^ we employ the method of Page and McIver.^12^ This method provides an exact solution to the differential equations of the path by employing a local quadratic approximation to the potential energy landscape at every step along the path. By following the path, two minima *A* and *B* can be connected to the transition state, ‡, lying between them and form a triplet of states {*A* ↔ ‡ ↔ *B*}.

### 2.7 Locating Stationary States on the Free Energy Landscape

The whole process described in the previous subsection operates under constant volume; i.e., all states of the triplet {*A* ↔ ‡ ↔ *B*} have the same spatial extent. However, since we are interested in transitions taking place under the condition of thermodynamic equilibrium in terms of free energy, we should allow the shape and volume of the system to be adjusted for every state in order to fulfill the equilibrium conditions, e.g., imposed lateral stress σ_⊥_. For the local minima, a detailed description of the two-level minimization strategy can be found in ref (13).

**Figure 1:**
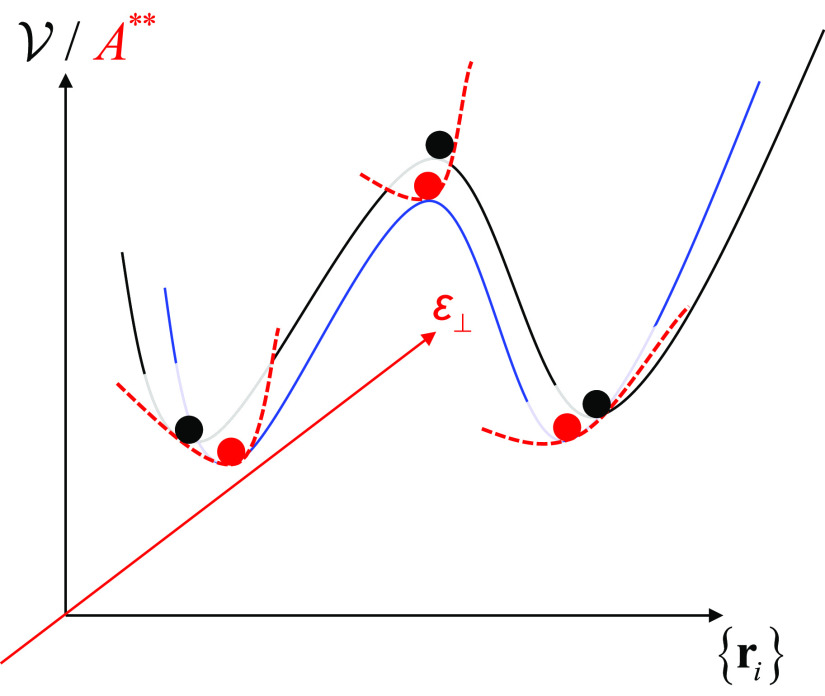
Free energy minimization in the vicinity of stationary points of the potential energy landscape (marked in black). A pair of potential energy minima connected through a first-order saddle point is depicted (black dots). For every one of them, an A** free energy minimization is conducted in order to drive the system to the corresponding free energy minima that are indicated by red dots. The underlying potential energy landscape following the free energy minimization is marked in blue. All states are characterized by the same principal strain ε and stress applied to the lateral dimensions, σ_⊥_.

As far as a potential energy transition state is concerned, we let the system minimize its free energy (either A** or *G*) under the constraint of keeping the structure of the Hessian, i.e., preserving its single negative eigenvalue. In essence, we minimize the free energy with respect to the shape and size of the simulation box, under the requirement that, for each set of box borders considered, the system remains constrained at a first-order saddle point of the potential energy. This is accomplished by changing the box dimensions, searching for a saddle point with the new dimensions by employing Baker’s algorithm^41^ for following the lowest eigenmode of the Hessian and then recalculating the free energy of the system contained within the updated domain. The vibrational free energy, *A*_vib_, is calculated for the configuration of the system at the saddle point, excluding one degree of freedom (the unstable one that corresponds to the negative eigenvalue). The process is summarized in Figure 1. In the following, we will study transitions states on the *G* and A** free energy landscapes. As far as the Gibbs energy, *G*, is concerned, the control variable will be the pressure *p*, and we will scan for changes in volume. As far as the A** free energy is concerned, we can control ε and σ_⊥_ and let the system obtain the ε_⊥_ that minimizes A**.

### 2.8 Free Energy Barrier and the Rate Constant

The knowledge of the triplet of connected states, {*A* ↔ ‡ ↔ *B*}, allows a rigorous calculation of the free energy of the transitions *A* → *B* and *B* → *A*, by means of the quasi-harmonic approximation that provides analytical estimates of the internal energy and entropy. Under an imposed stress tensor, **σ**, the strain tensor may be different between each state of the triplet. We choose to use the configuration at the transition state as the reference configuration with respect to which the strain tensor is calculated, i.e.,  = *V*^‡^; any of the minima can also be used. By employing the transition state as the reference configuration, the resulting expressions are fully analogous for both minima by exchanging the minimum index (*A* or *B*). In terms of the Gibbs energy, the *A* → *B* transition is inhibited by the free energy barrier:
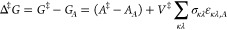
20where *V*^‡^ is the volume of the system at the transition state and ε_*κλ*_,_*A*_ is the strain tensor of configuration *A* with respect to the transition state. The  term introduced in eq 18 vanishes since both states employ the volume of the reference configuration. Since we use the configuration at the saddle point as the reference configuration for defining the strain tensor, the last term in the right-hand side of eq 20 appears with a positive sign. The corresponding enthalpic and entropic contributions can be easily obtained by
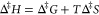
21and
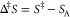
22At this point, we should note again that there is not a unique definition of enthalpy for a deformed solid specimen. We can define it as a familiar transform of *G* as in eq 21, but any other definition of an “enthalpy” function could be employed.^38^

In a completely analogous way, the barrier of the free energy A**, eq 14, for a transition taking place under imposed ε and σ_⊥_ is given by
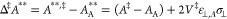
23where we have chosen the transition state as the reference configuration  and the lateral strain is calculated with respect to that configuration, i.e., ε_⊥_^‡^ = 0 (reference state). All states have the same (imposed) strain in the principal direction, i.e., ε_*A*_ = ε_*B*_ = ε^‡^, and the same stress is applied to the cross-section in the lateral directions. However, the strain in the cross-section, ε_⊥_, is different and obtained by the thermodynamic minimization process.

The rate constant for exciting state *A* through the transition state ‡ is equal to the ratio of the configurational integrals: one calculated over the dividing surface located at the transitions state divided by the one calculated over the entire basin corresponding to state *A*.^42^ For state-to-state transition, e.g., *A* → *B*, we should limit the configurational integral of the nominator to the part of the boundary surface of basin *A* that is common with the boundary surface of *B*. We can then consider the relevant partition functions, *Q*, instead of the configurational integrals, i.e.
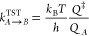
24where the Planck’s constant, *h*, takes care of the different dimensionalities of the relevant phase-spaces where the partition functions correspond to (for *Q*^‡^ we exclude the dimension normal to the dividing surface; the system is allowed to freely sample all other directions). In the case of applied pressure, e.g., in the *G* framework defined by eq 18, the transition rate constant becomes
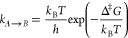
25which resonates with the familiar definition of the rate constant in isothermal–isobaric conditions. Similarly, the rate constant for transitions taking place under constant ε and σ_⊥_ is
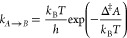
26implying that the partition functions in the  -ensemble are proportional to the Boltzmann factor of the A** free energy.

## 3 Results and Discussion

### 3.1 Finding a Transition State under Given ε, σ_⊥_

**Figure 2:**
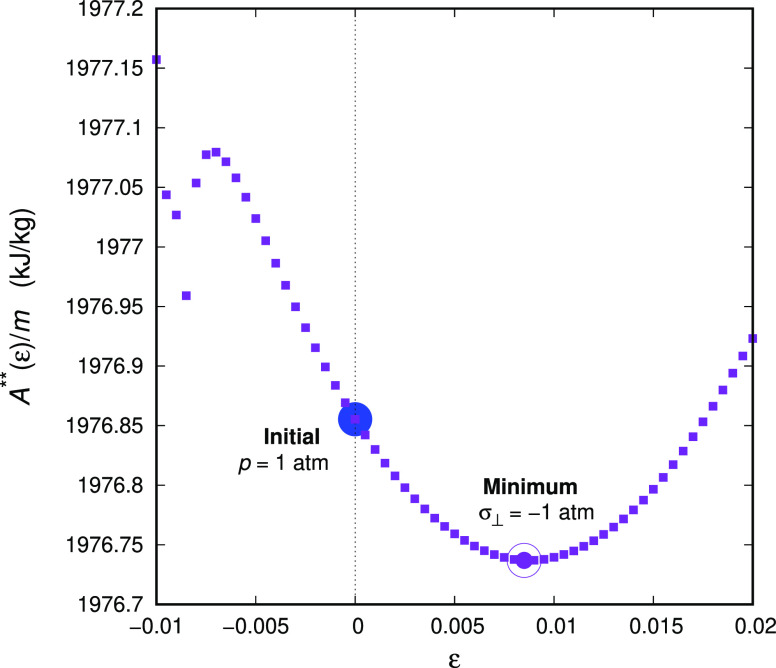
Free energy minimization in the glassy state. The blue circle indicates the configuration obtained after isobaric (*p* = 1 atm) quenching to the glassy state, i.e., the Gibbs energy minimum at temperature *T* = 300 K. The open magenta circle indicates the configuration corresponding to the free (A**) energy minimum, where the specimen is enforced to comply with pressure applied to the lateral crossection, while the principal direction of deformation is strain-controlled.

Quenching from the melt state under isobaric conditions (here *p* = 1 atm) results in the first configuration of the system in the glassy state. This resembles a freshly quenched specimen (after heating and annealing) locked in the basin of the energy landscape where it found itself upon cooling. In that case, the system is described by its Gibbs energy, *G*, and by minimizing *G* with respect to the components of the strain tensor under imposed hydrostatic pressure, we obtain a mechanically stable configuration in the glassy state, that is represented by the blue dot in Figure 2. This configuration is characterized by a density of ρ(300 K, 1 atm) = 1034 ± 9 kg/m^3^ which is in good agreement with the macroscopically observed density of polystyrene under the same conditions.^14^ Out of that configuration, we can initialize saddle point searches in order to discover transition states in the Gibbs energy landscape. In order to switch to the A** representation, we have to lock the cross-section of the specimen in two directions, i.e., constraining both directions to deform by the same strain, ε_⊥_, and search for an A** minimum. This is accomplished by scanning different principal strains, ε, and minimizing for every value of the principal strain (abscissa of Figure 2, we find the lateral strain ε_⊥_ that minimizes A** under given σ_⊥_). The procedure yields the configuration for which A** is minimal; i.e., any imposition of strain ε on the configuration increases the free energy. The resulting configuration is considered the reference configuration for given *T* and σ_⊥_. In our simulations, we keep the stress applied to the lateral cross-section fixed to −1 atm. The change of conditions, from equal pressure applied to all directions of the simulation box to the combination of a single strain-controlled dimension with equal pressure applied to the lateral cross-section, modifies the mechanical equilibrium of the specimen leading to different box dimensions (moving from the filled blue dot to the open magenta dot in Figure 2). The imposition of strain in the one direction lightly affects the density; in any case, the relative volumetric change, φ = 1 + *εε*_⊥_^2^ with respect to the configuration of minimal Gibbs energy is close to unity within 10^–5^.

**Figure 3:**
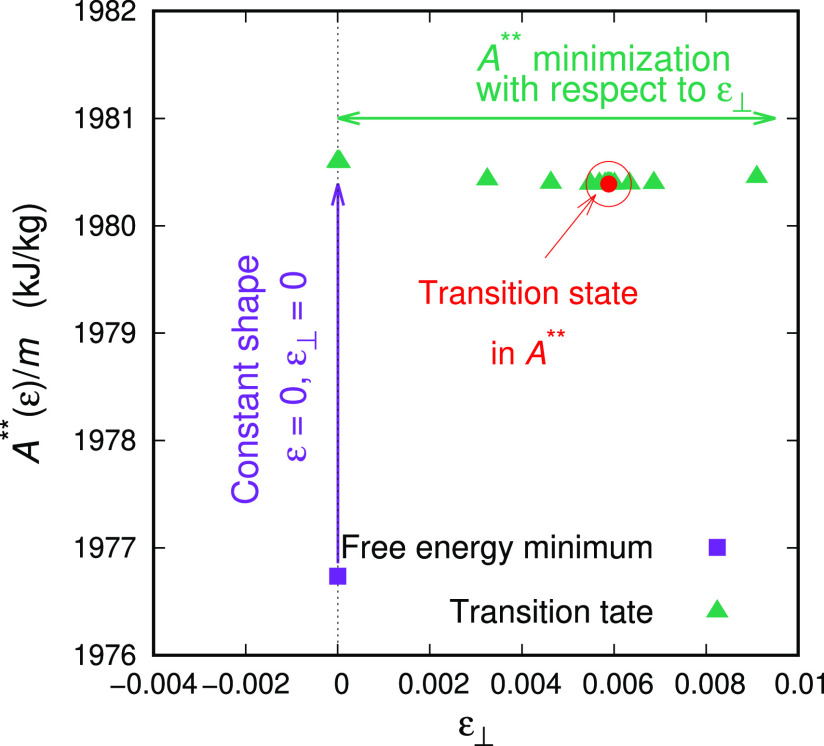
Saddle point search out of a free energy minimum. Initially, a first-order saddle point is discovered by stepping uphill under the constant shape of the simulation box^6^ (vertical magenta arrow in the figure). Once a transition state is reached, local free energy minimization is accomplished by optimizing the size of the lateral cross-section of the box (horizontal green arrow in the figure).

Once a free energy minimum, either in *G* or in the A** representation, is ensured, we set out to explore the energy landscape for first-order saddle points in the same representation. This is accomplished by a two-step procedure, as described in the Methodology section. First we try to locate potential energy saddle points by using the methods we developed in the past,^6^ and we then minimize the corresponding free energy at the transition state. The relevant procedure for finding transition states in the A** representation is presented in Figure 3. At first, the saddle point search is undertaken under constant shape and size of the simulation box represented by the vertical magenta arrow in Figure 3; i.e., the two strain measures ε (in the principal direction) and ε_⊥_ (in the lateral directions conjugate to the imposed stress σ_⊥_) are held constant. Once a transition state is found, we minimize the free energy by allowing the system to vary its lateral dimensions, i.e., by varying ε_⊥_, that is represented by the horizontal green arrow in Figure 3.

By splitting the problem of discovering saddle points in the free energy landscape into finding saddle points on the energy landscape and then changing their dimensions, the whole process becomes computationally tractable. However, it is important that a saddle point searching technique is used close to the transition state, instead for an energy minimization, since the latter may trigger a “fall-off” of the system to one of the neighboring potential energy minima. To this end, we employ the saddle point searching algorithm of Baker,^41^ tuned to follow the lowest eigenmode of the Hessian (corresponding to the single negative eigenvalue of the Hessian). Ideally, one could allow for free energy minimization with respect to ε_⊥_ at every step of the uphill path but that leads to an enormous computational cost. We have done that for a few configurations, and we found complete agreement with the scheme of imposing the free energy equilibrium only at the saddle point. Similar observations were also drawn by Kopsias and Theodorou.^24^ Once the saddle point on the free energy landscape is found, we initialize a minimal energy path exploration, following the quadratic method of Page and McIver^43^ for laying down the IRC on potential energy landscapes using a local quadratic approximation that we have also employed in our previous work.^6^ That leads us to the second minimum under a constant shape, that will also need ε_⊥_-optimization on the other side of the landscape. Finally, we end up with a triplet of states that are under the same thermodynamic constraints. In the case of the A** free energy, the triplet of the saddle point and the connected minima are stationary points of the A** hypersurface; i.e., they are characterized by the same ε and σ_⊥_, but different strain in the cross-section, ε_⊥_.

### 3.2 Response to Temperature under Constant Pressure

We start by studying the temperature dependence of the free energy barriers, Δ^‡^*G*. This is accomplished by minimizing the Gibbs energy of every triplet of connected states (saddle point and minima) with respect to the components of the strain tensor under prescribed atmospheric pressure at different temperatures. We start from the reference temperature of *T* = 300 K, where our previous work is conducted,^6^ and we either cool down or heat up the systems. The size of the simulation box is allowed to change, obeying the externally imposed pressure, *p* = 1 atm. There are two different ways of obtaining the configurations of the minima at different temperatures. The first approach is the direct heating or cooling of the minima from the initial to the target temperature (allowing their domain size to change appropriately). The other approach is heating or cooling of the saddle point and then constructing the IRC path from the saddle point to the neighboring minima under constant temperature. Both methods provide identical configurations for the free energy minima, and we thus make no distinction from now on.

**Figure 4:**
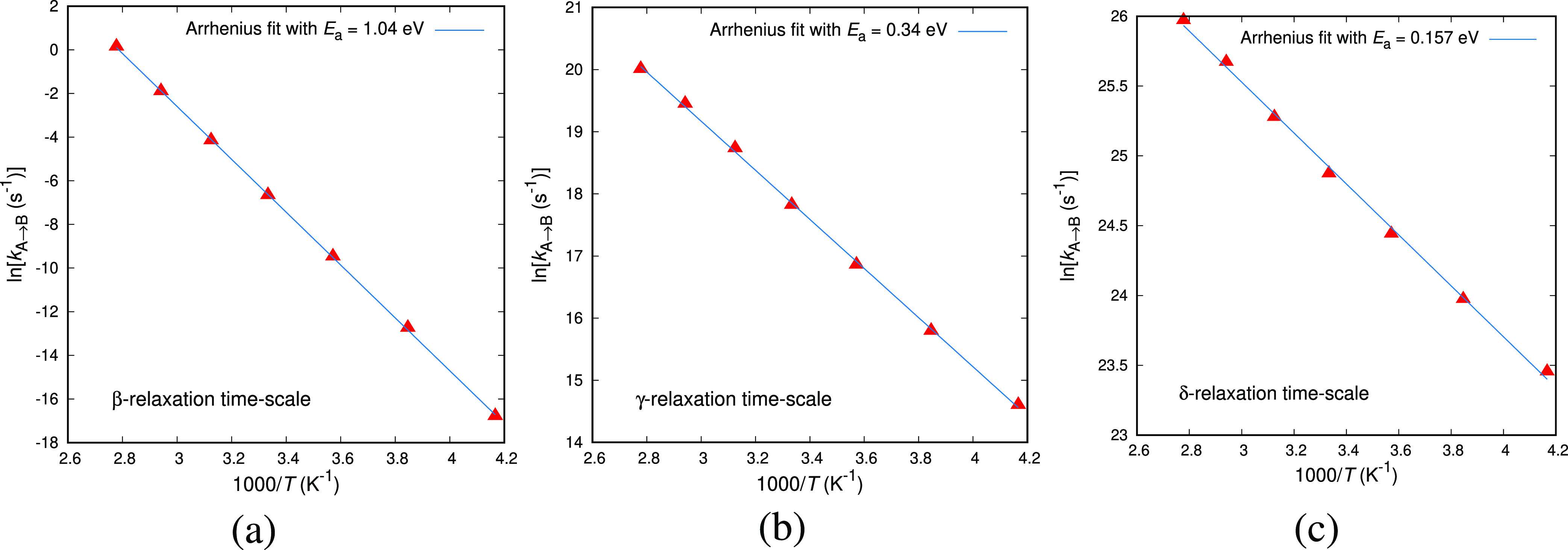
Arrhenius plots of the rate constants of individual transitions, whose rate constants at *T* = 300 K lie within the time-scales of the (a) β-, (b) γ-, and (c) δ-relaxations of atactic polystyrene, respectively. Gibbs energy minimization has been conducted under *p* = 1 atm for every temperature. The straight lines are best fits to an Arrhenius equation with activation energy *E*_a_.

The rate constant dependence on temperature for specific transitions is presented in Figure 4. Following the procedure described above, we study the response to temperature changes of our ensemble of transition states. Out of the ensemble of transition states presented in Figure 10 of ref (6), we choose those with rate constants at *T* = 300 K in the proximity of the macroscopically observed subglass relaxations. Three individual transitions are examined in Figure 4, each one corresponding to the β-, γ-, and δ- relaxations of atactic polystyrene. For all transitions we observe linear behavior with temperature that can be described well by an Arrhenius law. The activation energy of the best fit Arrhenius equation is also reported. The values obtained experimentally will be discussed in the following paragraphs. The temperature dependence of the rate constants of all elementary transitions sampled are fitted by an Arrhenius equation; we have not found elementary transitions, i.e., basin-to-basin displaying a strong non-Arrhenius character. This is in par with the experimental observations that all subglass relaxations exhibit an Arrhenius character, in contrast to the α-relaxation, the latter being a complex relaxation mechanism, where the system follows a sequence of elementary transitions and the macroscopic relaxation is a superposition of elementary events.

**Figure 5:**
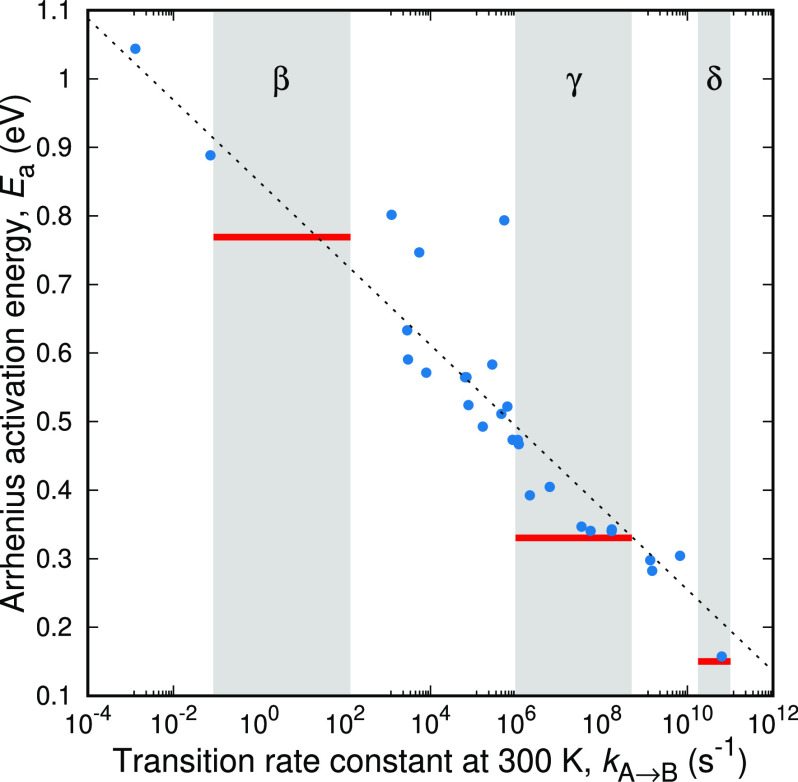
Activation energy of the different transitions studied versus the rate constant of every transition at *T* = 300 K. The gray bands represent the experimentally observed subglass relaxations of atactic polystyrene and the red horizontal lines the corresponding experimentally obtained activation energies. The dashed line has a slope of −*k*_B_*T*.

By taking this a step further, we perform temperature sweeps (cooling and heating) on all transition pairs available. For every transition, we fit the dependence of the rate constant on temperature to an Arrhenius law, and we store the activation energy. We then group the transitions by their rate constant at 300 K; the results are presented in Figure 5. The simulation results (solid circles) cover a range of activation energies from 0.15 to 1.1 eV, with the activation energy of individual elementary structural transitions increasing with a decreasing rate constant (or increasing relaxation time). Alongside the simulation results, we mark with gray bands the range of relaxation times associated with the subglass relaxations of polystyrene as those were obtained experimentally (gray bands in Figure 5). Moreover, we use the red straight lines to indicate the experimental estimate of the activation energy for every process. The scaling of the activation energy with the logarithm of the rate constant is indicated by the black dashed line, i.e., . Most transitions within the studied time-scales seem to conform to Arrhenius activated processes. There are, however, few outliers which do not seem to follow the general trend.

The characteristics of the δ-relaxation are obtained by the neutron scattering experiments of Arrese-Igor et al.,^44^ while the characteristics of the γ- and β-relaxations are obtained from the dielectric spectroscopy experiments (for a sample freshly quenched from the melt) of Grigoriadi et al.^45^ We see that there is very good agreement between simulation and experiment. However, instead of clear-cut bands of rate constants, corresponding to the macroscopic relaxation mechanisms, molecular simulations provide a continuous spectrum of rate constants, i.e., transitions being scattered around the straight dashed line in Figure 5. Even the experimental measurements of the macroscopic manifestation of the segmental relaxations indicate an overlap of the different relaxation mechanisms.^44^

**Figure 6:**
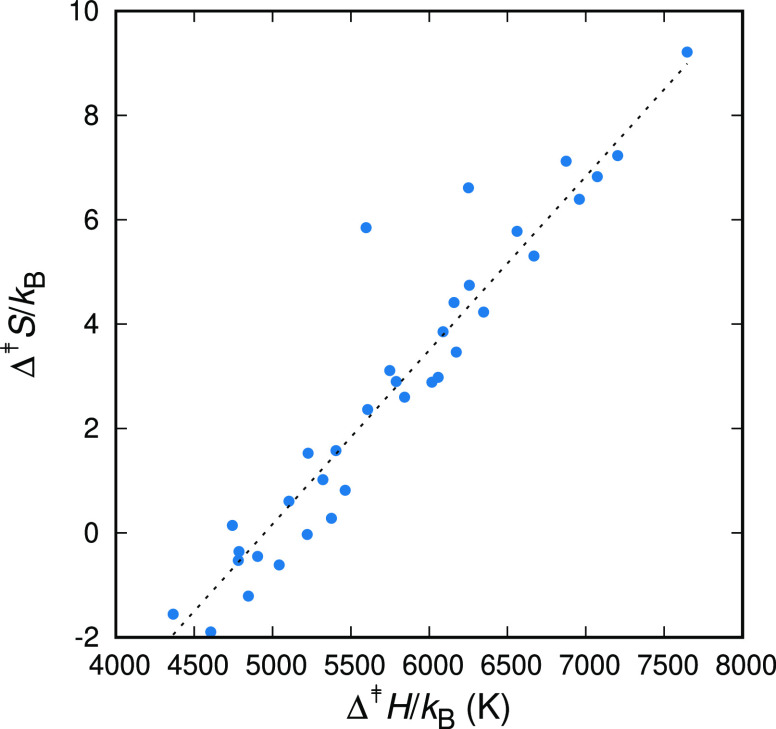
Entropy of activation, Δ^‡^*S*, versus the enthalpy of activation, Δ^‡^*H*, for all transitions exhibiting rate constants on the order of the inverse time-scale of the γ-relaxation. The selection of transitions was done on the basis of *T* = 300 K, and the chosen transitions were heated and cooled under atmospheric pressure, *p* = 1 atm. The dashed line has a slope of 1/*T* (see text for details).

An experimentally observed characteristic of the γ-relaxation of polystyrene is the complementary roles of enthalpy and entropy; i.e., transitions characterized by high enthalpic barriers exhibit also high entropic barriers, known as “enthalpy–entropy compensation EEC”.^46−48^ The general applicability of this empirical law is still controversial. In our simulations, we can sort the transitions at room temperature, based on their corresponding rate constants, and group those that correspond to the time-scales of an experimentally observed relaxation mechanism. In Figure 6 we present the height of the entropic barrier, Δ^‡^*S*, versus the height of the enthalpic barrier, Δ^‡^*H*, for the elementary transitions that can be assigned to the γ-relaxation. By following this procedure, we can clearly observe a linear increase of the height of the entropic barrier with the height of the enthalpic one. The same happens for groups of transitions corresponding to the other relaxation mechanisms (δ- and β-). However, this observation could be anticipated within our simulation framework. By grouping the transitions by means of their rate constant, that corresponds to a specific free energy barrier, a compensation between enthalpy and entropy should come into play for the overall rate constant to be within the limits set, since Δ^‡^*G* = Δ^‡^*H* – *T*Δ^‡^*S*. The most important feature of Figure 6 is the fact that the range in Δ^‡^*S* (and thus Δ^‡^*H*) values is considerable at *T* = 300 K.

### 3.3 Response to Pressure under Constant Temperature

In the following we are studying the pressure dependence of the relaxation time-scale of a transition, whose rate constant falls into the range of γ-relaxation. Intrinsic to the pressure dependence of transition rates (or segmental relaxation times) is information related to the apparent activation volume of the relaxation process:^49,50^
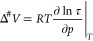
27where the relaxation time can be interpreted as τ = 1/*k*. Harmandaris and co-workers^50^ have followed a similar analysis in the melt state of PS; their simulations indicated an apparent activation volume of 400 cm^3^/mol for the lowest temperature of 350 K they studied. That volume referred to the segmental dynamics of PS, and it is probably too large for the activated motions in the glassy state. In our case, by studying the response to pressure of individual transitions, we can correlate the activation volume to specific rate constants; i.e., we can rigorously associate the dynamics of the molecular motion with the portion of the specimen that participates in the transition, or alternatively the length-scale of the rearrangement.

**Figure 7:**
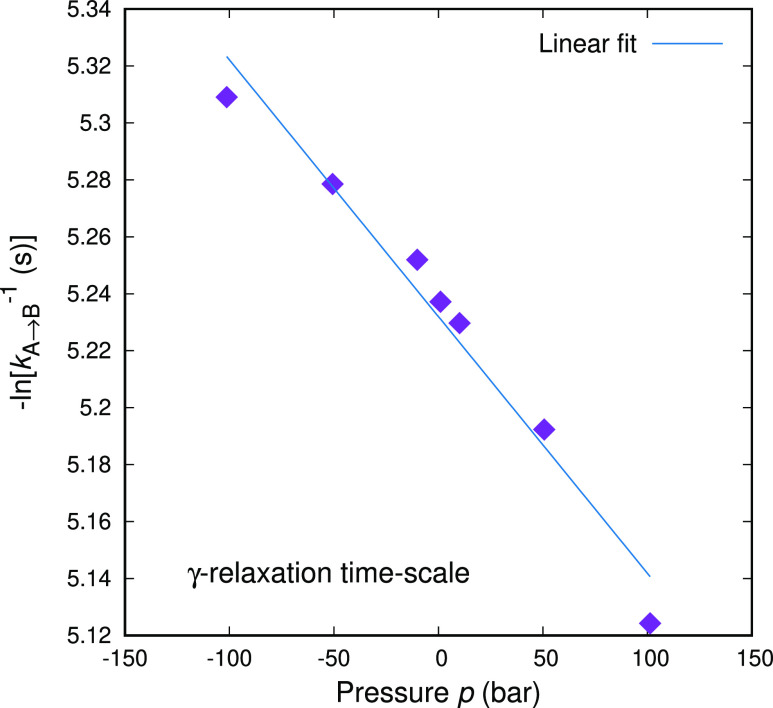
Pressure dependence of the rate constant of an elementary structural transition whose rate constant at *T* = 300 K and *p* = 1 atm lies within the γ-relaxation regime. The ordinate of the figure follows the convention introduced by Floudas and Reisinger.^49^

The pressure dependence of an individual transition, whose rate constant corresponds to the range of the macroscopic γ-relaxation at *T* = 300 K and *p* = 1 atm, is presented in Figure 7. The rate constants have been obtained by compressing and dilating the transition state (at different levels of hydrostatic pressure) and recreating the IRC at every pressure level. The new minima reached by IRC are then allowed to minimize their Gibbs energy under the same pressure. For that specific relaxation, we can estimate the apparent activation volume via eq 27: Δ^*#*^*V* = 22.475 cm^3^/mol. For the same temperature and pressure, the volume of styrene monomer can be estimated as *V*_mon_(*T* = 300 K, *p* = 1 atm) ≃ 101 cm^3^/mol. The comparison between the two volumes indicates that only a small fraction of a styrene monomer participates in this specific transition. While the molecular origin of the γ- relaxation is still unclear, our findings lie on par with the latest neutron scattering interpretation of the transition as local low-amplitude motions rather than full flips of the phenyl rings.^44^

### 3.4 Response to Imposed Strain in One Direction

**Figure 8:**
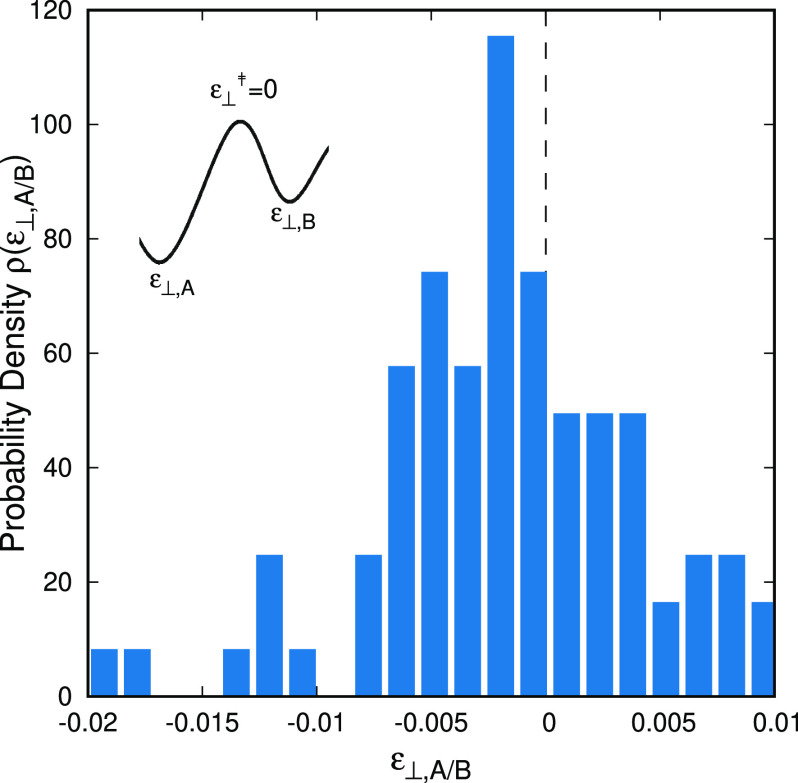
Distribution of strain of free energy minima with respect to the transition states at which the IRC calculation was initiated. In the inset to the figure a simple schematic presents the definition of the strain in connection to the discussion in Section 2.8. All stationary points of the free energy are not deformed in the principal direction, i.e., ε = 0.

An interesting feature observed in the case of saddle point searching under constant pressure is that saddle points are slightly diluted to allow for reorganization of the polymer during the transition through them. By changing from the Gibbs energy representation to the A** free energy representation, we, more or less, artificially constrain the expansion of the system in the principal direction of deformation by determining the principal strain ε. If the system would assume macroscopic dimensions, its size under the conditions of (*T*, *p*) would be the same as its size under . However, this is not the case we showed in Figure 2 due to the discrete nature of the matter at the length-scales of molecular simulations.^26^ Transition states in the A** landscape and their connected minima are characterized by the same ε but different ε_⊥_. The distribution of the strain in the lateral directions with respect to the configuration of the transition state (that is considered reference ε_⊥_^‡^ = 0) is presented in Figure 8.

The differences in the lateral strains are small, on the order of 10^–2^ at most, but clearly present. We observe that the main part of the distribution is located on the negative semiaxis. A transition state involves an elementary structural rearrangement of the system. For that rearrangement to take place, the system should allow some free space; i.e., the dimensions of the system at the saddle point should be slightly larger (or equivalently the strain imposed to arrive at the minima negative). However, there is a fair part of it that is found on the positive semiaxis, indicating actual contraction of the saddle points with respect to the minima. We consider that this should probably be an artifact of the finite-sized simulation domains we are employing. Despite being dense and consisting of a few thousands of atoms, our simulations systems are still extremely small by macroscopic criteria and far from being homogeneous and uniform. Moreover, a transition occurring locally within a small region will become less important if the size of the studied system becomes larger. Even by considering the maximum contraction of the system by ε_⊥,*A*/*B*_ = −0.02, the corresponding “work”, carried out by the system (due to its contraction) while it moves from the saddle point to the minimum, *W* = *V*^‡^ε_⊥_σ_⊥_≃ 0.005 kcal/mol, is only a minute fraction of the thermal energy at the same temperature, *k*_B_*T* ≃ 0.6 kcal/mol.

**Figure 9:**
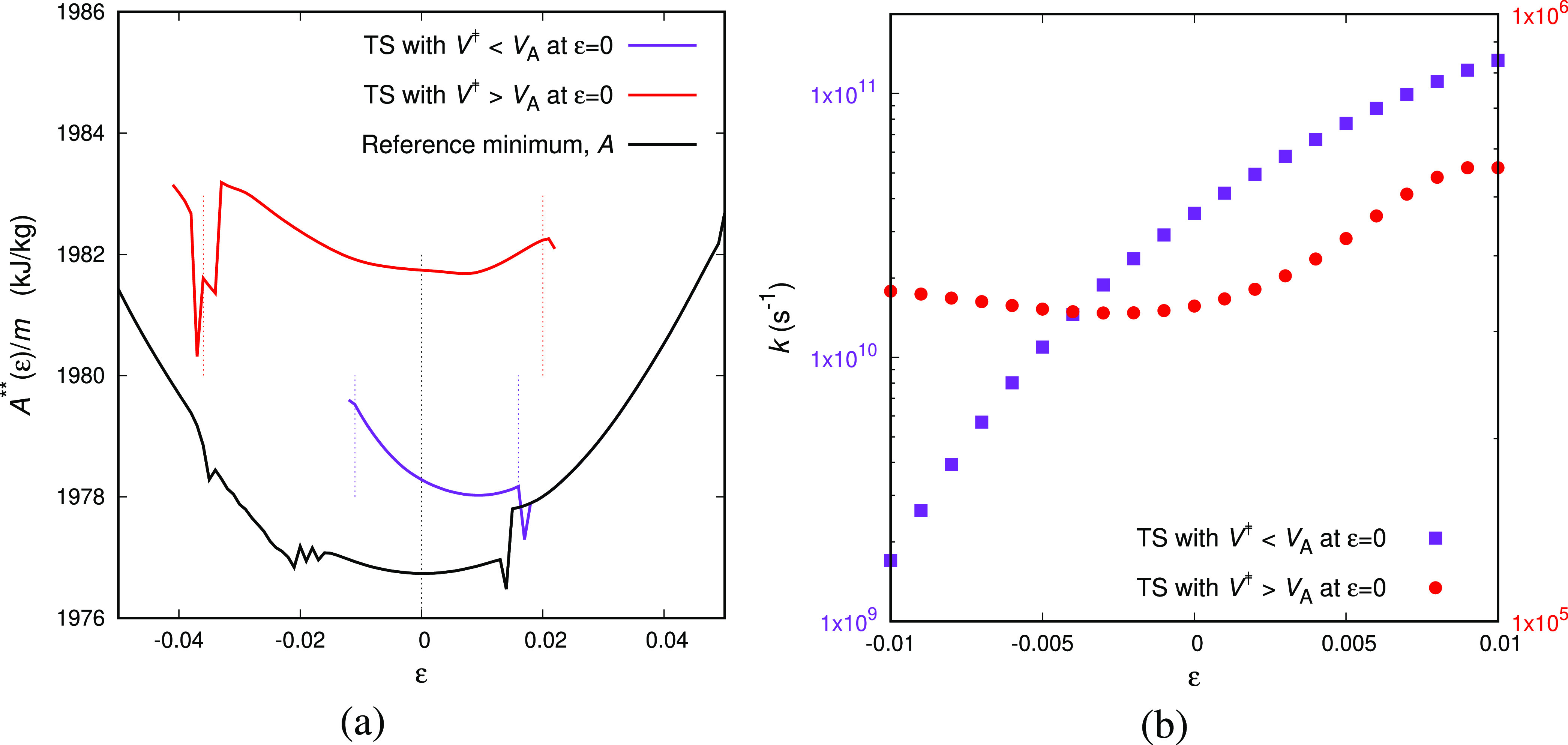
Response of several transitions states, linked to the same reference local minimum, to deformation. (a) Free energy energy as a function of strain for uniaxial extension/compression. (b) Transition rate as a function of the applied strain in the loading direction. All deformation simulations are performed at *T* = 300 K.

Having set the framework for strain-controlled uniaxial deformation experiments, we can now set out to explore the response to deformation of a free energy minimum and its surrounding transition states. In complete analogy to our discussion concerning the Gibbs energy above, there are two ways of obtaining the transition states as a function of imposed deformation. We can either deform the initial minimum and initiate new saddle point searches at every point along the deformation path, or search for saddle points for ε = 0 and then deform the saddle points by following the same protocol as the minimum. For all three transition states included in Figure 9, we have followed both routes that proved fully equivalent, up to the limits of stability of the transition states, indicated by the dashed vertical lines in the figure.

We can probably discern three groups of transition states for a given minimum in terms of their response to imposed strain ε. Since we impose ε, the only way to check the dimensions is by monitoring ε_⊥_. The first group includes the transition states that have their free energy minimum at the same dimensions as the minimum, i.e., ε_⊥,*A*/*B*_ ≃ 0 (we should note the convention of using the transition state as the reference configuration of every triplet of states). The second is the group of transition states whose volume is smaller than the volume of the neighboring minima, *V*^‡^ < *V*_*A*/*B*_ at ε = 0, thus ε_⊥,*A*/*B*_ > 0, like the purple line in Figure 9a. As we deform this group of transition states (by imposing ε), they yield a free energy saddle point at higher ε than the free energy minimum (e.g., around ε ≃ 0.01 for the purple line of Figure 9). For this group of transitions, we can reasonably anticipate that the rate constant increases upon extension and decreases upon compression, see also Figure 9b, since the parabolic free energy curve of the saddle point is translated to higher ε with respect to the parabolic free energy curve of the minimum. Finally, there is a third group of transition states that would produce the opposite behavior; i.e., the transition states are characterized by higher volumes than the neighboring minima, *V*^‡^ > *V*_*A*/*B*_ or ε_⊥,*A*/*B*_ < 0. We anticipate that this group of transitions is the most important one, since the transition states are slightly diluted allowing for easier rearrangement of the atoms.

The dependence of the transition rate constant on strain is complicated since it depends on the relative position (on the ε-axis) of the minimal free energy of the potential energy minimum and the potential energy saddle point. The overall balance of the tendencies of the rate constants would probably give rise to a “macroscopic” Eyring-type dependence of rate constant on strain and eventually stress. It can be obtained only after averaging all of the aforementioned groups of transition states, and the immediate connection of the microscopic measurements like Figure 9b to simplified macroscopic models is still elusive.

**Figure 10:**
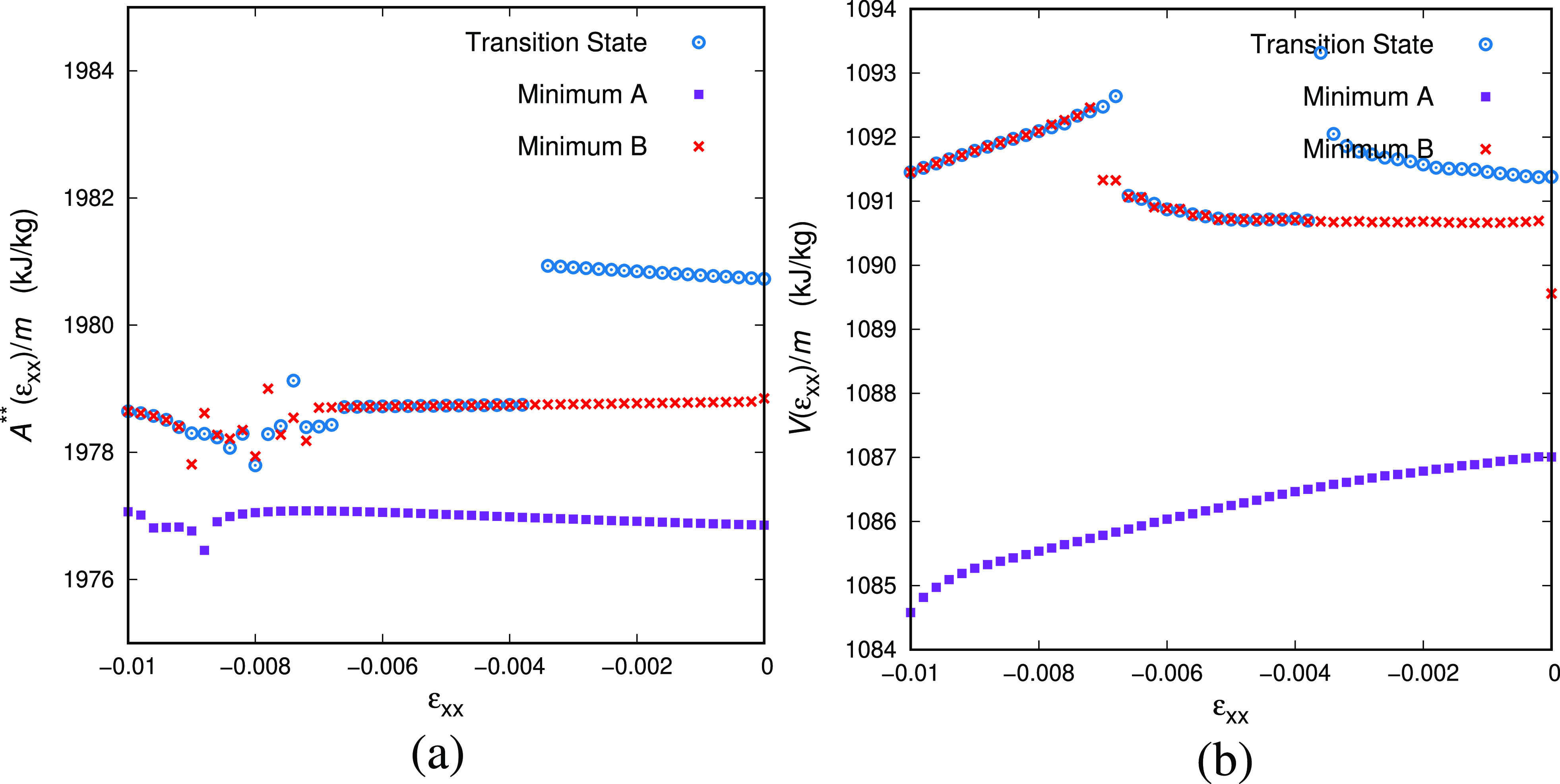
Response of triplet of connected minima and transition state to deformation. (a) Helmholtz energy as a function of strain for pure uniaxial extension/compression. (b) Potential energy as a function of strain.

The last point to deal with is the mechanism of collapse of transition states onto the connected minima as a result of applied deformation. Figure 10 presents the behavior of the free energy and the potential energy as a function of imposed strain for a triplet of connected states. Initially, we have a triplet of connected states, depicted by the free energy curves of different colors in Figure 10a. The transition state is initially well-separated from the two minima. However, upon compression, we observe that the transition state disappears abruptly, collapsing onto one of the two minima. This event is not smooth, even after decreasing the step-size in strain used for the free energy calculations. We speculate that there are two reasons for this sudden disappearance of the saddle point. First, the system contains a complex molecular architecture encompassing large side groups that behave as rigid domains upon deformation, giving rise to irreversible (plastic) events, e.g., the flip of a phenyl ring. Massive events of this kind are extremely sensitive to the local environment shaped by the deformation and give rise to sharp discontinuities. Another reason contributing to the disappearance of stationary points may be the finite size of our simulation systems. If we could allow a macroscopically sized (in the limit of length-scales that matter can be treated as a continuum) simulation box to change shape in a continuous way (imposing one element of the strain tensor and allowing all other elements of the strain tensor to change independently), we might be able to observe a smooth transition from the saddle point to the neighboring minima. This is a point of ongoing research.

## 4 Conclusions

The response to temperature and imposed deformation of transition states corresponding to elementary structural rearrangement events were studied for atactic PS specimens. A combination of methods allowed us to sample triplets of connected states (pairs of minima connected to each other via first-order saddle points) on the free energy landscape shaped by the externally imposed constraints. All states were under the same mechanical conditions (e.g., strain imposed in the loading direction and pressure applied to the cross-section in the lateral directions). The coupling of an efficient saddle point searching method^6^ to a framework for performing minimization of free energy functionals^13^ enables the transformation of the potential energy landscape to a free energy landscape for any system described by classical molecular force fields. This provides a shortcut to the, otherwise intractable, problem of exploring a free energy landscape where both the mircroscopic (atomic coordinates) and macroscopic (simulation box geometry) conditions change simultaneously. Our framework is rigorous and can predict properties that are experimentally accessible.

As far as the elementary transitions on the free energy landscape of PS are concerned, we were able to unravel a wealth of information. The sampled transitions follow Arrhenius dependence on temperature to a very good approximation; the relevant activation energies were found to be in good agreement to the available experimental estimates. For transitions in the range of the macroscopic γ-relaxation of PS, a compensation relation between enthalpy and entropy was observed, in line with recent experimental findings.^51^ The dependence of transition rates on the pressure provided us with an estimate of the size of the regions of the material that participate to the transition. For the subglass relaxations (δ- and γ-), the size of the participating regions was smaller than the average size of a polymer repeat unit. Overall, for every elementary structural transition (through a transition state that is a first-order saddle point), we accessed both its time-scale (and its dependence on temperature) and its length-scale (by estimating the apparent activation volume of the transition). By imposing uniaxial deformation instead of pressure, we could study the response of the transition states to the imposed strain and tried to connect our findings to empirical laws of the strain dependence of transition rates. However, this is a formidable task since each individual elementary transition behaves in a completely different way. Any macroscopic behavior could only be obtained by extensive averaging over a vast ensemble of different transitions. Finally, the collapse of transition states to their neighboring minima as a result of their deformation was observed to be an abrupt discontinuity on the free energy landscape. There is still a plethora of unresolved problems, e.g., the effect of temperature on the deformation pattern of a triplet of connected states (two local minima communicating over a transition state), or the extension of the methods to thin films, which are going to be addressed in future studies.

## References

[ref1] Goldstein M. Viscous liquids and the glass transition: a potential energy barrier picture. J. Chem. Phys. 1969, 51, 3728–3739. 10.1063/1.1672587.

[ref2] Debenedetti P. G.; Stillinger F. H. Supercooled liquids and the glass transition. Nature 2001, 410, 259–267. 10.1038/35065704. 11258381

[ref3] Stillinger F. H.; Weber T. A. Hidden structure in liquids. Phys. Rev. A 1982, 25, 978–989. 10.1103/PhysRevA.25.978.

[ref4] Stillinger F. H.; Weber T. A. Dynamics of structural transitions in liquids. Phys. Rev. A 1983, 28, 2408–2416. 10.1103/PhysRevA.28.2408.

[ref5] Theodorou D. N.; Suter U. W. Atomistic modeling of mechanical properties of polymeric glasses. Macromolecules 1986, 19, 139–154. 10.1021/ma00155a022.

[ref6] Vogiatzis G. G.; van Breemen L. C. A.; Hütter M. Structural Transitions in Glassy Atactic Polystyrene Using Transition-State Theory. J. Phys. Chem. B 2021, 125, 7273–7289. 10.1021/acs.jpcb.1c02618. 34161106 PMC8279558

[ref7] Fukui K. Formulation of the reaction coordinate. J. Phys. Chem. 1970, 74, 4161–4163. 10.1021/j100717a029.

[ref8] Fukui K. The path of chemical reactions - the IRC approach. Acc. Chem. Res. 1981, 14, 363–368. 10.1021/ar00072a001.

[ref9] Mousseau N.; Barkema G. T. Traveling through potential energy landscapes of disordered materials: The activation-relaxation technique. Phys. Rev. E 1998, 57, 2419–2424. 10.1103/PhysRevE.57.2419.

[ref10] Munro L. J.; Wales D. J. Defect migration in crystalline silicon. Phys. Rev. B 1999, 59, 3969–3980. 10.1103/PhysRevB.59.3969.

[ref11] Baker J.; Chan F. The location of transition states: A comparison of Cartesian, Z-matrix, and natural internal coordinates. J. Comput. Chem. 1996, 17, 888–904. 10.1002/(SICI)1096-987X(199605)17:7<888::AID-JCC12>3.0.CO;2-7.

[ref12] Page M.; McIver J. W. On evaluating the reaction path Hamiltonian. J. Chem. Phys. 1988, 88, 922–935. 10.1063/1.454172.

[ref13] Vogiatzis G. G.; van Breemen L. C.; Theodorou D. N.; Hütter M. Free energy calculations by molecular simulations of deformed polymer glasses. Comput. Phys. Commun. 2020, 249, 107008 10.1016/j.cpc.2019.107008.

[ref14] Lempesis N.; Vogiatzis G. G.; Boulougouris G. C.; van Breemen L. C.; Hütter M.; Theodorou D. N. Tracking a glassy polymer on its energy landscape in the course of elastic deformation. Mol. Phys. 2013, 111, 3430–3441. 10.1080/00268976.2013.825018.

[ref15] Mols R. H. M.; Vogiatzis G. G.; van Breemen L. C. A.; Hütter M. Microscopic Carriers of Plasticity in Glassy Polystyrene. Macromol. Theory Simul. 2021, 30, 2100021 10.1002/mats.202100021.

[ref16] Mott P. H.; Argon A. S.; Suter U. W. Atomistic modelling of plastic deformation of glassy polymers. Philos. Mag. A 1993, 67, 931–978. 10.1080/01418619308213969.

[ref17] Eyring H. Viscosity, Plasticity, and Diffusion as Examples of Absolute Reaction Rates. J. Chem. Phys. 1936, 4, 283–291. 10.1063/1.1749836.

[ref18] Malandro D. L.; Lacks D. J. Relationships of shear-induced changes in the potential energy landscape to the mechanical properties of ductile glasses. J. Chem. Phys. 1999, 110, 4593–4601. 10.1063/1.478340.

[ref19] Malandro D. L.; Lacks D. J. Molecular-Level Mechanical Instabilities and Enhanced Self-Diffusion in Flowing Liquids. Phys. Rev. Lett. 1998, 81, 5576–5579. 10.1103/PhysRevLett.81.5576.

[ref20] Lacks D. J. Energy Landscapes and the Non-Newtonian Viscosity of Liquids and Glasses. Phys. Rev. Lett. 2001, 87, 225502 10.1103/PhysRevLett.87.225502. 11736406

[ref21] Chung Y. G.; Lacks D. J. Atomic mobility in strained glassy polymers: The role of fold catastrophes on the potential energy surface. J. Polym. Sci., Part B: Polym. Phys. 2012, 50, 1733–1739. 10.1002/polb.23166.

[ref22] Maloney C. E.; Lacks D. J. Energy barrier scalings in driven systems. Phys. Rev. E 2006, 73, 061106 10.1103/PhysRevE.73.061106. 16906808

[ref23] Vogiatzis G. G.; Megariotis G.; Theodorou D. N. Equation of State Based Slip Spring Model for Entangled Polymer Dynamics. Macromolecules 2017, 50, 3004–3029. 10.1021/acs.macromol.6b01705.

[ref24] Kopsias N. P.; Theodorou D. N. Elementary structural transitions in the amorphous Lennard-Jones solid using multidimensional transition-state theory. J. Chem. Phys. 1998, 109, 8573–8582. 10.1063/1.477522.

[ref25] Lyulin A. V.; Michels M. A. J. Molecular dynamics simulation of bulk atactic polystyrene in the vicinity of Tg. Macromolecules 2002, 35, 1463–1472. 10.1021/ma011318u.

[ref26] Vogiatzis G. G.; Theodorou D. N. Local segmental dynamics and stresses in polystyrene–C60 mixtures. Macromolecules 2014, 47, 387–404. 10.1021/ma402214r.

[ref27] Born M.; Huang K. Dynamical Theory of Crystal Lattices; Oxford Classic Texts in the Physical Sciences; Clarendon Press: Oxford, UK, 1998.

[ref28] Flory P. J. Foundations of rotational isomeric state theory and general methods for generating configurational averages. Macromolecules 1974, 7, 381–392. 10.1021/ma60039a022.

[ref29] Go̅ N.; Scheraga H. A. On the use of classical statistical mechanics in the treatment of polymer chain conformation. Macromolecules 1976, 9, 535–542. 10.1021/ma60052a001.

[ref30] Theodorou D. N.; Suter U. W. Geometrical considerations in model systems with periodic boundaries. J. Chem. Phys. 1985, 82, 955–966. 10.1063/1.448472.

[ref31] Sangster M.; Strauch D. Localized modes associated with defects in ionic crystals. J. Phys. Chem. Solids 1990, 51, 609–639. 10.1016/0022-3697(90)90140-B.

[ref32] Tidor B.; Karplus M. The contribution of vibrational entropy to molecular association: The dimerization of insulin. J. Mol. Biol. 1994, 238, 405–414. 10.1006/jmbi.1994.1300. 8176732

[ref33] Andricioaei I.; Karplus M. On the calculation of entropy from covariance matrices of the atomic fluctuations. J. Chem. Phys. 2001, 115, 6289–6292. 10.1063/1.1401821.

[ref34] Weiner J. Statistical Mechanics of Elasticity; Wiley-Interscience: New York, 1983.

[ref35] Hetnarski R.; Eslami M. Thermal Stresses – Advanced Theory and Applications; Solid Mechanics and Its Applications; Springer Netherlands: Dordrecht, NL, 2008.

[ref36] Li J. C. M.; Oriani R. A.; Darken L. S. The Thermodynamics of Stressed Solids. Z. Phys. Chem. (Berlin, Ger.) 1966, 49, 271–290. 10.1524/zpch.1966.49.3_5.271.

[ref37] McLellan A. G.; Denbigh K. G. The chemical potential in thermodynamic systems under non-hydrostatic stresses. Proc. R. Soc. London, Ser. A 1968, 307, 1–13. 10.1098/rspa.1968.0170.

[ref38] Barron T. H. K.; Munn R. W. Thermodynamics of solids under stress. Pure Appl. Chem. 1970, 22, 527–534. 10.1351/pac197022030527.

[ref39] Morris J. W. Jr. Notes on the Thermodynamics of Solids; University of California: Berkeley, 2007; pp 366–411.

[ref40] Barkema G. T.; Mousseau N. Event-based relaxation of continuous disordered systems. Phys. Rev. Lett. 1996, 77, 4358–4361. 10.1103/PhysRevLett.77.4358. 10062518

[ref41] Baker J. An algorithm for the location of transition states. J. Comput. Chem. 1986, 7, 385–395. 10.1002/jcc.540070402.

[ref42] Truhlar D. G.; Garrett B. C.; Klippenstein S. J. Current Status of Transition-State Theory. J. Phys. Chem. 1996, 100, 12771–12800. 10.1021/jp953748q.

[ref43] Page M.; Doubleday C.; McIver J. W. Following steepest descent reaction paths. The use of higher energy derivatives with ab initio electronic structure methods. J. Chem. Phys. 1990, 93, 5634–5642. 10.1063/1.459634.

[ref44] Arrese-Igor S.; Arbe A.; Frick B.; Colmenero J. Glassy dynamics of polystyrene by quasielastic neutron scattering. Macromolecules 2011, 44, 3161–3168. 10.1021/ma2001178.

[ref45] Grigoriadi K.; Putzeys T.; Wubbenhorst M.; Breemen L. C. A.; Anderson P. D.; Hutter M. Effect of low-temperature physical aging on the dynamic transitions of atactic polystyrene in the glassy state. J. Polym. Sci., Part B: Polym. Phys. 2019, 57, 1394–1401. 10.1002/polb.24883.

[ref46] Dyre J. C. A phenomenological model for the Meyer-Neldel rule. J. Phys. C: Solid State Phys. 1986, 19, 5655–5664. 10.1088/0022-3719/19/28/016.

[ref47] Yelon A.; Movaghar B. Microscopic explanation of the compensation (Meyer-Neldel) rule. Phys. Rev. Lett. 1990, 65, 618–620. 10.1103/PhysRevLett.65.618. 10042969

[ref48] Yelon A.; Movaghar B.; Crandall R. S. Multi-excitation entropy: its role in thermodynamics and kinetics. Rep. Prog. Phys. 2006, 69, 1145–1194. 10.1088/0034-4885/69/4/R04.

[ref49] Floudas G.; Reisinger T. Pressure dependence of the local and global dynamics of polyisoprene. J. Chem. Phys. 1999, 111, 5201–5204. 10.1063/1.479774.

[ref50] Harmandaris V. A.; Floudas G.; Kremer K. Temperature and Pressure Dependence of Polystyrene Dynamics through Molecular Dynamics Simulations and Experiments. Macromolecules 2011, 44, 393–402. 10.1021/ma102179b.

[ref51] McKenzie I.; Fujimoto D.; Karner V. L.; Li R.; MacFarlane W. A.; McFadden R. M. L.; Morris G. D.; Pearson M. R.; Raegen A. N.; Stachura M.; Ticknor J. O.; Forrest J. A. A β-NMR study of the depth, temperature, and molecular-weight dependence of secondary dynamics in polystyrene: Entropy–enthalpy compensation and dynamic gradients near the free surface. J. Chem. Phys. 2022, 156, 084903 10.1063/5.0081185. 35232192

